# Sex differences shape the response to infectious diseases

**DOI:** 10.1371/journal.ppat.1006688

**Published:** 2017-12-28

**Authors:** Molly A. Ingersoll

**Affiliations:** 1 Unit of Dendritic Cell Immunobiology, Department of Immunology, Institut Pasteur, Paris, France; 2 INSERM U1223, Institut Pasteur, Paris, France; Nanyang Technological University, SINGAPORE

## Sex is a neglected variable in infectious disease

Historically, we have overlooked sex as a variable in infectious disease research [1, 2]. For example, while much of our knowledge comes from animal studies, many researchers routinely use only male animals [3]. One of the principal reasons for this is the argument that female animals, undergoing cyclic hormonal fluctuations, introduce additional experimental variation [4]. Sex bias is also a major challenge in clinical studies. In 1977, Food and Drug Administration (FDA) guidelines for human studies recommended that women of reproductive age be excluded from early clinical trials (e.g., Phase I) [1, 5]. While more recent efforts have resulted in greater inclusion of female subjects [5], the lasting consequence of this recommendation is that many drug regimens and therapeutic approaches are based solely on information gained from testing in male subjects [5–7]. Major adverse effects experienced by female patients underline that single-sex studies cannot predict whether and how men and women will respond differently to a drug, vaccine, or treatment [7].

It has become increasingly clear that sex broadly influences the host immune response [1, 2, 8]. Indeed, the influence of sexual dimorphism is likely underappreciated. The analysis of more than 14,000 wild-type and 40,000 mutant mice revealed that approximately 10% of qualitative and more than 50% of quantitative phenotypes are influenced by sex in wild-type mice [9]. Similarly, mutant phenotypes were impacted by sex in approximately 13% of qualitative and 17% of quantitative traits analyzed [9]. At the gene expression level, modest but significant differences exist between male and female liver, adipose, muscle, and brain tissue in mice [10]. In humans, as in experimental animal systems, what we now appreciate is that men generally exhibit greater susceptibility, prevalence, and severity of infection compared with women, which can be seen across a wide variety of pathogens, including parasitic, fungal, bacterial, and viral infections [1, 2, 11, 12]. Exceptions to this generality, however, can be found in which susceptibility or severity to infection, for example, is more pronounced in women. Importantly, what drives these differences is still poorly understood. By taking a closer look at two examples, urinary tract infection (UTI) and influenza, we can begin to appreciate some of the many factors that likely drive these differences.

## Do hormones shape susceptibility to UTI?

UTIs have a distinctive pattern in that it is women who exhibit increased susceptibility and prevalence of infection, whereas men experience increased severity [13–15]. The prevalence of bacteriuria, or bacteria in the urine, is approximately 10% in adult women and 0.1% or 1/1000 men [16]. Why men experience UTIs less frequently is often attributed to anatomical differences between men and women, including urethra length [16]. However, several lines of evidence suggest that sex bias in UTI is driven not only by dissimilar urethra length [16] but by sex-based variation in the levels of specific hormones, such as testosterone or estrogen, between men and women over the course of a lifetime. For example, UTI incidence in male infants is nearly twice that of female infants, and in children under age 2, 40% of UTI patients are male [17, 18]. At the other end of the spectrum, the incidence of UTI in geriatric populations (>65 years) is roughly similar between men and women (14% in women vs 11% in men) [19]. Indeed, the sex difference in UTI is most pronounced in nongeriatric adults [13], coinciding with the highest levels of sex hormones. Thus, UTI risk and severity change over the lifetime of females and males, suggesting that sex hormone levels or other sex differences contribute to differing host responses.

Supporting this idea, the elimination of estrogen in an experimental setting by ovariectomy leads to higher bacterial burden following uropathogenic *Escherichia coli* infection compared with intact mice [20]. Estrogen supplementation augments expression of the antimicrobial gene human β-defensin 3 and strengthens urothelial junctions in vitro, which may positively impact barrier function in the bladder, protecting against infection [20]. Lastly, in a double-blind clinical study, topical estrogen application reduced the incidence of recurrent UTI in postmenopausal women, with benefit attributed to increased lactobacilli colonization and decreased vaginal pH [21]. Taken together, these findings suggest that estrogen may play a protective role against UTI, and its loss may make women more vulnerable to infection. Furthermore, if hormones shape susceptibility to (uro)pathogens, hormone manipulation may alter host immunity, and—in the case of UTI—potentially reduce incidence in women or both sexes. Additional preclinical and clinical research is needed to address the influence of estrogen and to explore this treatment avenue for UTI.

## Hormone manipulation alters the host response to influenza

Although supplemental estrogen appears to be protective in the case of UTI, hormone manipulation—such as contraceptive use or hormone replacement therapy—likely has a more nuanced impact on immunity. In an influenza model, direct comparison of the two sexes reveals that female mice exhibit greater morbidity and mortality than male mice, potentially because of elevated levels of cytokines, such as tumor necrosis factor (TNF)-α and C-C motif chemokine ligand 2 (CCL2), in female mice [22]. Interestingly, a reduction in hormone levels through gonadectomy decreases mortality in female mice and increases mortality in male mice [22]. When gonadectomized mice are supplemented with exogenous hormone, testosterone does not impact mortality in male mice, whereas estrogen signaling via estrogen receptor α leads to improved mortality [22]. This estrogen-mediated protection in female mice is dependent upon alterations in cytokine levels and the recruitment of neutrophils at later stages of infection [23]. Notably, estrogen supplementation results in very high levels of this hormone compared with intact, untreated female mice [22], suggesting that estrogen therapy may protect women against influenza; however, this remains to be tested.

Specifically in female mice, progesterone treatment decreases cytokine-mediated inflammation, induces the expansion of T helper 17 (T_h_17) T cells, and promotes accelerated lung tissue healing through the expression of the tissue repair molecule amphiregulin during primary influenza infection [24]. In addition to progesterone, the synthetic progestin analog levonorgestrel, used in oral contraceptives, limits morbidity while reducing serum antibody titers against a primary flu infection [25]. Despite reduced antibody titers, animals challenged with an influenza drift variant, encoding minor changes in sequence compared with the original virus, are protected regardless of the hormone treatment received [25]. By contrast, challenge with a heterologous influenza virus induces greater immune pathology and mortality, potentially mediated by decreases in virus-specific CD8^+^ T cells in progesterone- or levonorgestrel-treated mice compared with placebo-treated animals [25]. Together, these findings suggest that women using progesterone-based contraception may experience more severe responses to subsequent flu infection from season to season.

Finally, testosterone supplementation in aged male mice reduces clinical symptomology and mortality following influenza infection [26]. Interestingly, testosterone does not impact viral titer, pulmonary damage, or antibody production, leaving in question the exact mechanisms of its action in this model [26]. Taken together, given that the vast majority of women in the United States will use hormonal contraceptives at some point in their lifetime [27] and that hormone replacement therapy is used in many clinical settings in both men and women, these findings merit additional preclinical and human studies. The findings also support that treatment options for those suffering from infection should take into account not only the sex of the patient but their contraceptive and hormonal status.

## Nonhormonal sex-biasing differences influence host–pathogen interactions

Sex differences in infection can be mediated by more than hormonal influence [1]. The X chromosome expresses a number of immune-related genes, such as toll-like receptor 7 (TLR7) and Interleukin-1 receptor-associated kinase 1 (IRAK1), as well as a number of immune-associated microRNAs [28]. While X inactivation, or silencing of one X chromosome, in women would be expected to provide dosage compensation of X-linked genes, certain regions of the X chromosome escape inactivation [28, 29]. This can lead to higher transcription levels of specific genes, such as TLR7, leading to sex-specific responses to viral infection [28–30]. The Y chromosome also influences immune gene expression, regulation, and susceptibility to both noninfectious autoimmune diseases and infection [31]. For example, the Y chromosome mediates susceptibility to cocksackievirus independently of sex hormone expression [32]. Moving away from sex chromosomes, an analysis of eosinophil infiltration into lymph nodes following *Leishmania major* infection revealed that four autosomal loci control eosinophil numbers [33]. Of these loci, three appear to be influenced by sex, with one of the three regulating eosinophil infiltration only in infected male mice [33]. Additional work will be needed to determine the mechanisms behind these phenotypes.

## How can sex differences be more prominently addressed in research?

As diverse sex-based mechanisms clearly have a profound impact on disease susceptibility, severity, and response, the challenges of considering both sexes in infectious disease research must be addressed. The simplest step for researchers to take is reporting the sex of the animals, cells, or cell culture models used. The journal *Endocrinology* embraced this idea in 2012, specifying that the methods section of submitted manuscripts must indicate the sex of animals used or the sex of the animal from which primary cultures were derived [34]. Additional editors have advocated for the inclusion of sex reporting in submitted manuscripts; however, not all have mandated that this information is absolutely required [35]. Specifying the sex of the animal used, as well as clearly reporting whether only one sex was used in research studies, will highlight findings that may not be amenable to generalization to both sexes. Furthermore, some of the perceived reasons for excluding a particular sex may not be as relevant as originally thought. A recent meta-analysis of nearly 300 studies found that phenotypic variability is not greater in female animals compared with male animals, even when estrous cycle staging is not employed, dispelling the belief that female mouse studies are intrinsically more variable [36]. Efforts such as this analysis should help allay concerns and encourage researchers in fields that predominantly rely upon male animals (e.g., neuroscience, physiology, pharmacology, and endocrinology [3]) to consider female animal models.

Indeed, greater efforts to include male and female animals should be made when feasible or warranted. For example, with a single exception utilizing a surgical model of infection [37], no studies have directly addressed the sex bias in UTI. It is the opinion of several leaders in the field of sex-based differences that the inclusion of male and female animals in preclinical studies will ultimately lead to reduced costs and greater knowledge at the clinical stage [38]. Despite these obvious benefits, the inclusion of both sexes is not always an option because of constraints such as increased associated costs. Additionally, as research builds on published studies, findings that contradict the literature or reveal that specific phenotypes are not maintained in the opposite sex may face greater publishing challenges. While it will be difficult to overcome this type of challenge, specific mechanisms, such as the National Institutes of Health (NIH)’s administrative supplement for research on sex/gender influence, are aimed at supporting the increased costs associated with testing in both sexes (PA-17-078).

Finally, the inclusion of women in clinical trials has increased dramatically through the efforts of the FDA and NIH [5]. Policies such as the NIH Revitalization Act—recognizing that the exclusion of women from early-stage clinical trials has led to a deficit in the understanding of women’s health as well as sex-based differences—have emphasized that sufficient numbers of women must be included in clinical research and that studies should include specific analyses of sex-based differences [5]. The biggest challenge, however, is that many studies are not powered for separate analyses of men and women, which can lead to the erroneous conclusion that no differences exist between the sexes [35]. Going forward, efforts aimed at the inclusion of both sexes in animal and human studies, with sufficient power to analyze potential sexual dimorphism, will advance our understanding of host–pathogen interactions and lead to targeted therapies to safely combat infectious diseases in men and women.
